# Impact of working from home on European office rents and vacancy rates

**DOI:** 10.1365/s41056-022-00057-z

**Published:** 2022-04-04

**Authors:** Jaroslaw Morawski

**Affiliations:** University of Applied Sciences, Aschaffenburg, Germany

**Keywords:** Office Markets, Working from Home, Covid-19, C23, R33

## Abstract

The massive shift to working from home during the Covid-19 pandemic triggered discussions about its potential impact on the future demand for office space and the risk it poses to the performance of office markets. Against this background, the goal of this paper is to investigate the link between working from home and the evolution of key indicators of office occupier markets across Europe over the past three decades. Based on the data from Eurostat and CBRE, the paper uses panel regression to investigate the temporal as well as cross-sectional relationships between the share of the workforce working from home and office rents and vacancy rates in major cities. The results are interesting in several ways. Firstly, changes in the share of employees working from home did not appear to have any significant impact on the evolution of rents or vacancy rates over time. However, occasional homeworking was significant in explaining cross-sectional differences in office market indicators. Moreover, contrary to the initial expectations, higher share of employees occasionally working from home appeared to be associated with stronger performance of the respective office market. As explanation, the paper proposes a hypothesis that this was due to working from home being only one aspect of broader changes in the office work environment and related socio-economic trends that had a net beneficial effect on office occupier markets. Although the results refer to historical developments and may not be fully applicable to the current context of the pandemic, they highlight the need to consider working from home in a broader perspective of office occupier trends and ways of working.

## Introduction

One of the most visible impacts of the Covid-19 pandemic was the change in the daily routines of millions of office workers around the world. The lockdowns implemented in most countries resulted in a sudden shift of corporate activity from offices to employees’ homes, with office buildings either legally inaccessible or companies choosing this option in accordance with the official guidelines and to prioritise employee well-being. The unprecedented scale and the relative success of the global “working-from-home experiment” raised questions about a potential structural shift in the office sector. It has been argued that working from home may become a permanent solution for a large share of office occupiers, leading to collapsing demand for office space (Eavis and Haag 2021; Egan 2020). The discussion was further fuelled by announcements by some major companies regarding specific or potential plans to allow a large share of their employees to work from home on a regular basis (Deutsche Bank 2021; Kelly 2020; Microsoft 2020; Siemens 2020). While there is still a lot of scepticism about the long-term efficiency of homeworking solutions, if the trend continued, it could lead to a significant reduction of the demand for office space with the consequence of increasing vacancy, obsolescence and falling rents.

Amid the ongoing discussions, it is important to note that delivering office work from home is not a new idea. The phenomenon of shifting from a pure office environment to various modes of remote working has been ongoing for at least 20 years. Since corporate communication moved from paper to email, documents became instantly accessible online, and connection speeds and security improved, deep changes to the office working model have been inevitable. Even though the shift towards remote work was not as fast and revolutionary as some early predictions assumed, it was happening long before the pandemic.

It is still too early to indicate the direction of travel, and the outcome will depend on the evaluation of the lockdown experiences and subsequent corporate decisions in the coming years. However, even though an increase in working from home appears likely, its impact on the demand for office space is a separate question. Would office space occupied by a company decrease proportionally if a percentage of its staff worked from a different location? Recent discussions show that there is no consensus on this point. Some predictions, especially the early ones, indeed assume that offices will be used less, implying negative consequences for office markets (Cutter 2021; Nixey 2020). Others point out that the need for face-to-face communication remains, and it can be provided most efficiently in offices. Hence, even if some of the work was done from home, it would not have a major impact on the total amount of office space used by companies but merely change its character (Kröger 2021; Moran 2020; Schede 2021).

Against this background, the paper addresses the question of the sensitivity of office occupier markets to the share of employees working from home. However, the purpose is not to predict the outcome of the post-pandemic processes, but rather to look back and investigate, whether the past (slow) changes in the scale of homeworking had any measurable impact on the performance of office markets or allowed to explain international variation in market indicators. While the experience of the lockdowns was unique and may constitute a structural break that makes it difficult to project past experiences into the future, the historical evolution of markets can still contain some indications on the nature of the relationship between homeworking and aggregate office demand.

The issue can be approached from two distinct perspectives: the first one focusing on the evolution over time; the second one looking at cross-sectional differences between markets. Hence, two research questions arise:

1. Does a change in the share of home-workers lead to stronger/weaker performance of office occupier markets?
2. Do office markets with higher shares of home-workers tend to be stronger/weaker than markets with lower shares of home-workers?

The paper is organised as follows: Next section reviews the literature on the topic. It is followed by the discussion of pre-Covid trends with respect to working from home. Sect. 4 addresses the data and the methodology of the panel regression, while the results are presented in Sect. 5. The final section is devoted to the interpretation of the results and concluding remarks.

## Literature review

Literature on working from home is extensive and stretches over several decades. The early papers in the 1980s pointed out the possibilities of remote work that were arising from the introduction of personal computers to offices and homes (Olson and Primps 1984; Pratt 1984; Zuboff 1982). The subsequent main string of research focused on the work-life-balance and improvements to the well-being of employees. Numerous studies indicated that enabling workers to work from home could result in higher job satisfaction and improved productivity. This was, for example, the conclusion of the study by Bailyn (1989) conducted on a sample of UK computer firms. Numerous other studies reached similar conclusions, highlighting positive aspects of workplace flexibility (Bloom et al. 2013; Clancy 2020; Clark 2001; Duxbury et al. 1998; Fonner and Roloff 2010; Gajendran and Harrison 2007; Hill et al. 2003). On the other hand, various papers highlighted potential psychological issues arising from working from home, mostly related to the feeling of isolation and social detachment, lack of separation between work and private life, distraction and decreased commitment (Golden 2006; Harpaz 2002; Salomon and Salomon 1984). Consequently, an important research question was about the balance of advantages and disadvantages to individuals and companies and its dependence on various factors, in particular the characteristics of the individuals and their roles in the company (Allen et al. 2015; Ammons and Markham 2004; Bailey and Kurland 2002; Dockery and Bawa 2014; Golden and Veiga 2005; Harpaz 2002; Morganson et al. 2010; Olson 1983; Olson and Primps 1984). While most authors predicted a steady increase in working from home, the consensus was that it was a slow process that would stretch over years and decades, allowing sufficient time for demand and supply to adjust (Harris 2015).

Another, more recent, string of research focused on the assessments of the share of jobs that can be done from home. The widely cited paper by Dingel and Neiman (2020) uses the classification of occupations for that purpose; authors identify job characteristics that clearly rule out the possibility of working remotely. By combining it with US job statistics, they conclude that 37% of all jobs in the US could be done remotely; the share varies between 28% and 51% across US metropolitan areas. Another study by Adams-Prassl et al. (2020) goes further and investigates the tasks within occupations; it estimates the share of “remotable” jobs at 41% in the US and 39% in the UK. Other studies arrive to similar conclusions with the shares ranging mostly between 30% and 40% (Bartik et al. 2020; Cetrulo et al. 2020; Deng et al. 2020; Gottlieb et al. 2020; Holgersen et al. 2021; Lund et al. 2020).

Finally, a significant number of papers have been published in the recent months addressing the impact of the Covid-19 pandemic on working from home. Early studies were mainly descriptive, focussing on the scale of the phenomenon and the experiences of the employers and employees (Barrero et al. 2021; Brynjolfsson et al. 2020; Chung et al. 2020; Reuschke and Felstead 2020). The main research question was, however, if the “homeworking experiment” was a unique but temporary situation, or if working patterns were going to change permanently post-Covid. The vast majority of research appears to lean towards to latter thesis, with the consensus forming around a model with roughly 2 days per week spent at home and 3 days in the office (Alexander et al. 2021; Bloom 2020; Erdsiek 2021; Kamouri and Lister 2020; Kunze et al. 2020; Plößl and Just 2021; PwC 2021). This would be in line with the surveys conducted before the pandemic (Gallup 2017; Hood et al. 2018).

The other aspect addressed in this paper—performance of the rental markets for office space—received vast coverage in the literature in the past decades. From the occupier market perspective, (market) rent and vacancy rate are the key indicators determining the level of cashflows to owners. Numerous studies, both theoretical and empirical, demonstrated that these two indicators are closely linked: high vacancy creates a “tenant market” leading to weaker (negative) rent growth, while low vacancy is associated with a “landlord market” leading to stronger rent growth (Glascock et al. 1990; Hendershott et al. 1999; McDonald 2000; Wheaton and Torto 1988). Both rent growth and vacancy rate are therefore affected by similar supply and demand factors The latter include in particular GDP growth (D’Arcy et al. 1997; Gardiner and Henneberry 1989) and (office) employment (Dobson and Goddard 1992; Hendershott et al. 1999; Sivitanides 1997) as well as structural factors such as office space per employee (Miller 2014). It is through the employment variables, that a link between the performance of office markets and working from home can be suspected.

In the context of the potentially higher level of homeworking in the future, the long-term impact on the aggregate demand for office space and the performance of office markets continues to receive particular attention among practitioners. Most statements in the press so far reflect subjective views of real estate market participants, partially backed by anecdotal evidence. More rigorous analyses have been relatively few, focusing on the identification of office markets considered to be most at risk (DWS 2020) or on the design of a future office (Boland et al. 2020; JLL 2020). While several academic papers address the direct impact of the Covid-19 pandemic on commercial real estate values and returns (Balemi et al. 2021; Ling et al. 2020; Milcheva 2021; Voigtländer 2020), academic studies looking specifically into the long-term impact of increased working from home on office demand are still missing. This paper contributes to filling this gap.

## Pre-Covid-19 trends

As of 2021, it is still too early to judge what the future will bring for the office sector. However, looking back and analysing the past trends in homeworking can provide insights into the mechanisms involved in these processes. This section offers an overview of the state of homeworking in Europe in the decades preceding the Covid-19 pandemic.

Eurostat reports statistics on the share of employed persons working from home as a percentage of total employment (Eurostat 2021). This data is available annually since 1992 for 35 countries with some gaps especially in the early years. The dataset differentiates between those working from home usually, occasionally, and never. Based on this data, the share of employees (excluding self-employed) working occasionally from home in Europe began to increase around the turn of the century and has more than doubled from 3.3% in 2002 to around nearly 8% in 2020. At the same time, the share of employees working usually from home moved from 1.6% in 2002 to around 3% in 2019 and over 10% in 2020, reflecting a massive shift to working from home during the Covid-19 pandemic. These figures refer to all employees; the shares are obviously significantly higher for office workers compared to other jobs that require physical presence at the workplace. A global survey by GWA (Kamouri and Lister 2020) indicated that only 29% of office workers never worked from home. On the other hand, 9% worked from home 5 days a week, 22% worked from home 1–4 days a week, and 40% worked from home only occasionally. This mirrors the fact that most companies have been offering the option to work from home for some time now—more than 80% of companies surveyed in the Alternative Workplace Strategies Report (Hood et al. 2018) and virtually 100% of large enterprises. Hence, while full-time home working was rare, working from home on an occasional basis was reasonably widespread even before Covid-19.

**Fig. 1:**
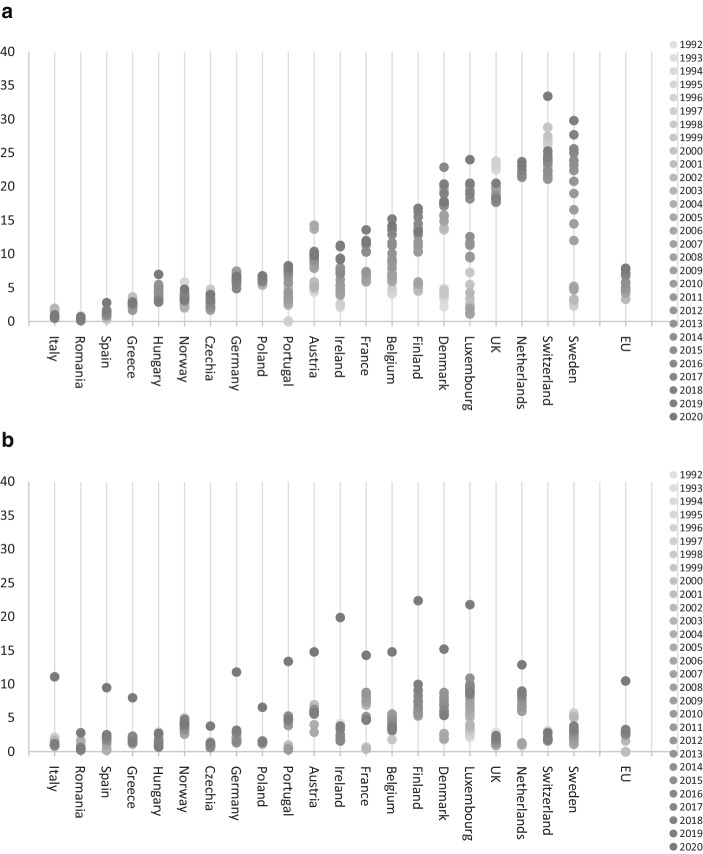
Share of employees working from home (% of employees excluding self-employed). **a** Working from home occasionally. **b** Working from home usually. (Source: Eurostat)

However, there was huge variation across countries as depicted in Fig. 1. The share of occasional home workers in 2019 ranged between less than 3% in Italy or Spain, to over 25% in the Netherlands or Sweden (referring to all employees). The gap between the top and the bottom ranking countries has widened considerably during the past 20 years. While the share of home-workers remained largely unchanged in Italy and Spain, it grew from 6% to 13% in France, and 3% to 30% in Sweden.

The significant differences in the adoption of remote office work across Europe are not easy to explain. It seems reasonable to assume that the technical possibilities are largely comparable in all countries. Hence, the differences appear to be rooted in the local corporate policies, cultural factors and personal situations of the population. It is striking that the share of home-workers tends to be higher in northern Europe than in southern Europe. This may correlate with the more open and communicative culture of southern Europeans. Another reason might be the availability of suitable workspace at home. According to Eurostat, 66% of Spanish and 52% of Italians live in apartments rather than in houses; the respective share is 33% in France and 19% in the Netherlands. According to Euroconstruct, the average dwelling area per inhabitant is 33 sqm in Spain and 37 sqm in Italy compared with 43 sqm in the Netherlands and 45 sqm in Sweden (Pajakkala 2018). A combination of these factors possibly limits the ability and desirability to work from home in southern Europe.

## Data and methodology

### Data

The study seeks to uncover the relationships between the key performance metrics of office occupier markets and the shares of the workforce working from home. The latter is measured using the data published by Eurostat and cited in the preceding section. However, the sample only covers the period until 2019 due to the specific impact of the lockdowns in 2020 resulting in that year being a clear outlier. The Eurostat data refers to the share of home-workers among all employees, not all of whom can work from home, which means that it likely underestimates the share of office workers working from home. However, since the analysis focuses on changes over time and comparisons across markets, potential biases in levels should not invalidate the results.

With respect to the office market variables, the paper focuses on two indicators: percentage annual changes of prime office rents and office vacancy rates; this data has been provided by CBRE. Since the Eurostat dataset is on the national level, and the office market metrics are on the city level, each country is matched with the major office market in this country. In most cases, only one clearly dominating office market exists—this is for example the case for the UK (London) and France (Paris). However, in Germany several cities have significant office stock and economic weight. It is common to speak of the Big 5 markets that include Berlin, Dusseldorf, Frankfurt, Hamburg, and Munich. In this case, the office market data is averaged across these five locations.

The sample includes annual data covering the period 1992–2019 for 29 countries and cities for which both office market data and homeworking statistics are available. However, even this data contains gaps, so that the effective sample sizes are ranging between 324 and 484, depending on the specification of the model.

Real GDP growth reported by Eurostat is used to control for the economic environment. As discussed in Sect. 2, it is recognised as one of main drivers of office market performance in numerous studies and as such should capture fundamental economic factors not related to working from home.

### Methodology

The paper uses an unbalanced panel regression to analyse the relationship between homeworking and the selected metrics of office markets. This method is preferable to a regression based on pooled data or individual year-by-year regressions as it allows utilising both cross sectional and time series properties of the sample. Furthermore, it gives the opportunity to control for the impact of omitted variables through the option of using fixed or random effects (Hsiao 2007). Using cross-section fixed effects is analogical to using a country dummy variable and hence assuming that the impact of the explanatory variables over time is country specific. With period fixed effects, it is assumed that there is a varying impact of an unobservable variable in each period of the cross-section category. This impact is estimated as a vector of constants that capture the temporal dimension of the process. Hence, regression coefficients refer only to the cross-sectional dimension in this case. In contrast, random effects assume that the variation is attributable to random realisations of the same variable. For example, using random cross-section effects is equivalent to assuming that the variation across countries is attributable to random realisations of the same underlying market cycle and there are no country-specific temporal trends. To determine whether fixed or random effects are more appropriate, the likelihood ratio test can be used to verify if fixed effects were redundant while the Lagrange multiplier test can be used to check for omitted random effects.

Estimating the panel model with cross-section and period effects allows examining the relationship in two different contexts. In the first context, the focus is on the temporal dynamics, and the research question can be formulated as: does an increase in homeworking lead to an increase/decrease in office rents or vacancy rates? In this case, cross-sectional fixed effects proved to be redundant, and the Lagrange multiplier test suggested the use of cross-section random effects, which implies the existence of a pan-European property cycle common to all markets. In the second context, the focus is on the impact of homeworking across countries, addressing the question: do office markets in countries with higher shares of homeworking tend to see stronger/weaker rent growth and/or higher/lower vacancy rates than markets in countries with lower shares of employees working from home? The likelihood ratio tests suggested that period fixed effects were appropriate in this case.

For models based on cross-section, heteroskedasticity may be a potential issue. Unfortunately, no test is available for a panel regression. However, to counter this potential problem, heteroskedasticity-robust standard errors (White cross-section standard errors & covariances) were used to counter possible heteroskedasticity in the panel data (Arrelano 1987). Furthermore, since two variables refer to homeworking in the same model—shares of occasional and usual home-workers—co-linearity could be a potential problem. We verified it using centred Variance Inflation Factors (VIFs). With centred VIFs well below 5, we concluded that co-linearity was at an acceptable level, even when certain subjectivity in evaluating VIF levels was considered (O’Brien 2007). Finally, the relationships between variables may involve delayed reactions. We tested for this eventuality by re-running the models with lagged variables and concluded that they led to a much lower explanatory power for models with cross-section fixed effects (temporal perspective) and very similar results for models with period fixed effects (cross-sectional perspective).

## Panel regression results

The analysis consists of two parts, the first part focusing on temporal effects and the second part focussing on cross-sectional effects. Referring to the research questions formulated in Sect. 1, the parts address the first and the second question, respectively. Since the paper looks at two office market indicators, rent growth and vacancy rate, two variants of each research question result. Operationalising them yields following four questions to be answered with the panel regression:

### 1a.

Does a change in the share of home-workers lead to stronger/weaker rent growth?

### 1b.

Does a change in the share of home-workers lead to increasing/decreasing vacancy rates?

### 2a.

Do markets with higher shares of home-workers tend to see stronger/weaker rent growth?

### 2b.

Do markets with higher shares of home-workers tend to see higher/lower vacancy rates?

### Temporal perspective

Referring to the first research question (1a and 1b), this part of the analysis focuses on temporal dynamics. The dependent variable is respectively the annual percentage rent growth and the change in the vacancy rate, while the independent variables are first differences in the shares of employees working from home occasionally or usually. Furthermore, to control for the impact of market environment, GDP growth is included in the regression. Since differences between countries are less relevant in this setting, they are neutralised by using cross-sectional effects. As explained in Sect. 4.2, using random effects proved to be more appropriate in this case.

The results presented in Table 1 indicate that neither the share of usual home-workers nor the share of occasional home-workers had a significant impact on rent growth. The model has low explanatory power with an adjusted R‑squared of only 0.13, but is highly significant as a whole based on the F‑Test. While the Durbin-Watson statistic at 1.37 is lower than ideally desired, it does not yet indicate significant issues with the autocorrelation of residuals.

**Table 1:** Panel regression of rent growth on WFH with cross-section random effects

Dependent Variable: D(PRIME_RENT)/PRIME_RENT(-1) Total panel (unbalanced) observations: 454 White cross-section standard errors & covariance (d.f. corrected) Cross-section random effects				
Variable	Coefficient	Std. Error	t‑Statistic	Prob
C	−0.007034	0.008802	−0.799080	0.4315
D(WFH_USUALLY)	0.002232	0.004147	0.538151	0.5951
D(WFH_OCCASIONAL)	−0.001763	0.002193	−0.803887	0.4288
GDP	0.012752	0.002371	5.378906	0.0000***
*R‑squared*	*0.133063*	*F‑statistic*		*23.02295*
*Adjusted R‑squared*	*0.127283*	*Prob(F-statistic)*		*0.000000*
		*Durbin-Watson statistic*		*1.371410*

Stars indicate significance levels of 1%(*), 5%(**) and 10%(***)

The results for the impact of homeworking on changes in vacancy rates are summarised in Table 2 and are very similar. Neither of the homeworking variables is significant. Also in this case, the explanatory power is low, even though the model is significant, and the Durbin-Watson statistic of 1.34 indicates mild autocorrelation of residuals.

**Table 2:** Panel regression of vacancy changes on WFH with cross-section random effects

Dependent Variable: D(VACANCY_RATE) Total panel (unbalanced) observations: 344 White cross-section standard errors & covariance (d.f. corrected) Cross-section random effects				
Variable	Coefficient	Std. Error	t‑Statistic	Prob
C	0.746437	0.410598	1.817927	0.0806
D(WFH_USUALLY)	0.027370	0.164032	0.166857	0.8688
D(WFH_OCCASIONAL)	−0.030040	0.102045	−0.294384	0.7708
GDP	−0.370597	0.078917	−4.696057	0.0001***
*R‑squared*	0.176836	*F‑statistic*		14.21151
*Adjusted R‑squared*	0.169119	*Prob(F-statistic)*		0.000000
		*Durbin-Watson statistic*		1.337569

Stars indicate significance levels of 1%(*), 5%(**) and 10%(***)

### Cross-sectional perspective

The second part of the analysis addresses the second research question (2a and 2b) and focuses on the differences between markets. The homeworking variables used in the model are identical as in the first part of the analysis, but they are used in levels rather than first differences. This is because the question of interest is whether the relative level of homeworking can explain the differences in office market indicators. The variation over time that may be driven by cyclical factors is neutralized by applying period fixed effects.

The results presented in Table 3 indicate, that the share of occasional home-workers positively impacted rent growth at a confidence level of 5%, while the impact of regular homeworking was not significant. The model has a moderate explanatory power with an adjusted R‑squared of 0.29 and is highly significant as a whole based on the F‑Test.

**Table 3:** Panel regression of rent growth on WFH with period fixed effects

Dependent Variable: D(PRIME_RENT)/PRIME_RENT(-1) Total panel (unbalanced) observations: 472 White cross-section standard errors & covariance (d.f. corrected) Period fixed effects (dummy variables)				
Variable	Coefficient	Std. Error	t‑Statistic	Prob
C	−0.003170	0.007957	−0.398398	0.6936
WFH_USUALLY	0.000882	0.001427	0.617952	0.5420
WFH_ OCCASIONAL	0.001025	0.000444	2.307207	0.0293**
GDP	0.006697	0.001852	3.616085	0.0013***
*R‑squared*	*0.334048*	*F‑statistic*		*7.645210*
*Adjusted R‑squared*	*0.290354*	*Prob(F-statistic)*		*0.000000*

Stars indicate significance levels of 1%(*), 5%(**) and 10%(***)

The results for the impact of homeworking on vacancy rates are summarised in Table 4. They are similar but less clear-cut than those for rent growth and indicate that the impact of occasional homeworking was significantly negative at 10%, while the share of usual home-workers had no significant impact. Also in this case, the regression model has a moderate explanatory power and is significant as a whole.

**Table 4:** Panel regression of vacancy rate on WFH with period fixed effects

Dependent Variable: VACANCY RATE Total panel (unbalanced) observations: 351 White cross-section standard errors & covariance (d.f. corrected) Period fixed effects (dummy variables)				
Variable	Coefficient	Std. Error	t‑Statistic	Prob
C	11.37471	1.037842	10.95996	0.0000
WFH_USUALLY	−0.364671	0.233367	−1.562648	0.1331
WFH_ OCCASIONAL	−0.126043	0.070790	−1.780522	0.0895*
GDP	−0.128557	0.102026	−1.260045	0.2215
*R‑squared*	*0.304852*	*F‑statistic*		*4.677796*
*Adjusted R‑squared*	*0.239682*	*Prob(F-statistic)*		*0.000000*

Stars indicate significance levels of 1%(*), 5%(**) and 10%(***)

## Interpretation of the results and conclusions

### Implications

The panel regression presented in the previous section yielded several noteworthy results.

Firstly, when looking at the temporal dynamics, none of the homeworking variables appeared to have any measurable impact on office markets. While the review of the Eurostat data in Sect. 3 already let suspect that this might be the case for homeworking on a regular basis, which was low and changed little over time, the increase in occasional homeworking was very substantial in some countries. As it seems, this increase did not translate into any meaningful over- or underperformance of the respective office markets. One possible explanation is that occasional working from home did not remove the need for an office, so that it did not eventually affect the aggregate office demand. Another possible explanation is that the slow nature of the changes allowed the supply side to adjust before any potential change in office demand could affect rents or vacancy rates.

Secondly, contrasting with the first result, occasional working from home proved to be significant in explaining international variation in market indicators. Indeed, the differences in the working patters across countries were quite big, especially towards the end of the analysed period, and apparently big enough to affect the relative performance of office markets. While the evolution of homeworking over time may have been too slow to result in measurable changes of rents or vacancy rates, contemporaneous comparison of markets with different characteristics revealed a much stronger relationship.

The third major result is significantly more astonishing and refers to the direction of the impact. The coefficient for the share of employees occasionally working from home was significantly positive in the rent growth model and significantly negative in the vacancy rate model in the cross-sectional perspective. In other words, it seems that a higher level of working from home was associated with a stronger rent growth and a lower vacancy rate of the corresponding occupier market. This is in stark contrast with the initial intuition as well as with the bulk of the discussions among academics and practitioners, some of whom argue that the increasing trend towards homeworking, accelerated by the Covid-19 pandemic, represents a risk that could render parts of the office stock obsolete and have an adverse impact on occupancy and rent levels. Based on the results in this paper, historical trends appear to be pointing in the opposite direction.

As counter-intuitive as the last result may appear, it leads to the recognition that the relationship between working from home and office demand or market performance is not straightforward. It should not be regarded as a simplistic equation of less work in the office = less demand for office space. In fact, the most likely explanation of this result is that working from home was not a stand-alone phenomenon but rather linked with other differences in the styles of office work and related socio-economic backgrounds in different countries. The analysed period 1992–2019 was marked by the introduction of technology to the workplace, but also by important social trends, such as increased focus on work-life-balance and health aspects. At the same time, innovation and collaboration gained increased attention. Hence, flexibility in the choice of the workplace was potentially only one aspect in the set of broader changes in the office work environment that had an overall net beneficial effect on office markets, being effectively a reflection of more mature corporate cultures. This view is also in line with much of the literature reviewed in Sect. 2 that often highlighted the multi-faceted nature of working from home.

The final conclusion refers to the highly significant impact of GDP growth on rent growth and changes in vacancy rates and is much less controversial. In fact, it is in line with most of the existing research on drivers of office markets, as discussed in Sect. 2. Indeed, it appears intuitive that the fundamental strength of the economy should be one of the main drivers of office demand and consequently of occupier market performance. The only setting in which GDP growth proved to be not significant was the model explaining cross-sectional variation in vacancy rates (Table 4), which was likely due to structural differences between office markets rather than cyclical factors.

### Limitations

While interpreting the results of the analysis, one needs to bear in mind the limitations arising from the available data. Firstly, the Eurostat survey on working from home does not differentiate between different types of jobs. Obviously, not all jobs can be done from home and not all of them can be done in offices. Secondly, the data on working from home is only available on the national level, while office market indicators refer to cities that may follow somewhat different trends. Since the analysis in this paper refers to relativities between markets and changes over time, these inaccuracies should not affect the validity of the results as such but it may lead to some biases for individual markets.

Another limitation may arise from using a very broad demand-related controlling variable—the national GDP growth—and not including a specific supply variable. The latter was not available for most markets for most of the analysed period, and including it would greatly reduce the sample. Since the impact of new supply is usually delayed, it should matter less in the analysis of contemporaneous cross-sectional relationships, but it may be more relevant in the time-series perspective. In particular, it may be one of the reasons why the historical increase in working from home did not appear to affect occupancy or rents—the slow changes in demand due to increased homeworking could have allowed enough time for office stock to adjust.

The last aspect may be very different in the context of the Covid-19 pandemic, when changes predicted to take many years or even decades had to be implemented within weeks. Therefore, the results of the analysis in this paper should not be treated as a dismissal of the risk to the office markets, especially in the short-term. Much more, they call for further research to gain deeper understanding of the complex processes associated with increased work from home and their wider impact on property markets.

### Future research

The most immediate direction for further research on the topic at hand is the inclusion of further office market variables. Subsequent iterations of the analysis could focus on the relationships between working from home and the evolution of office occupier markets in terms of detailed demand and supply trends, going beyond prime rents and aggregate vacancy rates.

In the medium term, an obvious direction for further research will be the analysis of Covid-19 related effects. Given the huge attention that the changing working patterns received during the prolonged period of the pandemic, much more detailed data will be available in the near future. In this context, it will be essential for both academics and practitioners to investigate in how far these changes were only a temporary consequence of the pandemic, or whether they can be generalized as an extension and acceleration of the trends present already before 2020.

Finally, an interesting field for research arises from the surprising conclusions of this paper regarding the positive relationship between the scale of working from home and the relative strength of the office markets. The hypotheses offered here is that this result was due to homeworking not being a separate trend but rather an aspect of broader differences in (office) working environments. Future research could verify this statement by including additional labour market variables such as working culture, flexible working hours or health and well-being aspects. This direction of research would provide a deeper understanding of the demand trends that have been affecting office markets in the recent years and will most likely continue to affect them in the future.
